# Body mass index interacts with a genetic-risk score for depression increasing the risk of the disease in high-susceptibility individuals

**DOI:** 10.1038/s41398-022-01783-7

**Published:** 2022-01-24

**Authors:** Augusto Anguita-Ruiz, Juan Antonio Zarza-Rebollo, Ana M Pérez-Gutiérrez, Esther Molina, Blanca Gutiérrez, Juan Ángel Bellón, Patricia Moreno-Peral, Sonia Conejo-Cerón, Jose María Aiarzagüena, M Isabel Ballesta-Rodríguez, Anna Fernández, Carmen Fernández-Alonso, Carlos Martín-Pérez, Carmen Montón-Franco, Antonina Rodríguez-Bayón, Álvaro Torres-Martos, Elena López-Isac, Jorge Cervilla, Margarita Rivera

**Affiliations:** 1 Department of Biochemistry and Molecular Biology II, Faculty of Pharmacy, University of Granada, Granada, Spain; 2 Institute of Nutrition and Food Technology “José Mataix”, Biomedical Research Center (CIBM), University of Granada, Granada, Spain; 3 Instituto de Investigación Biosanitaria ibs.GRANADA, Granada, Spain; 4 CIBEROBN (Physiopathology of Obesity and Nutrition CB12/03/30038), Institute of Health Carlos III (ISCIII), Madrid, Spain; 5 Institute of Neurosciences ‘Federico Olóriz’, Biomedical Research Center (CIBM), University of Granada, Granada, Spain; 6 Department of Nursing, Faculty of Health Sciences, University of Granada, Granada, Spain; 7 Department of Psychiatry, Faculty of Medicine, University of Granada, Granada, Spain; 8 Primary Care District of Málaga-Guadalhorce, Biomedical Research Institute of Málaga (IBIMA), Primary Care Prevention and Health Promotion Network (redIAPP), Málaga, Spain; 9 Department of Public Health and Psychiatry, Faculty of Medicine, University of Málaga, Málaga, Spain; 10 San Ignacio Health Centre, Primary Care Research Unit, Osakidetza Bilbao, Spain; 11 Federico del Castillo Health Centre, Jaén, Spain; 12 Parc Sanitari Sant Joan de Déu, Fundació Sant Joan de Déu, Barcelona, Spain; 13 CIBERESP, Centro de Investigacion Biomedica en Red de Epidemiologia y Salud Publica, Madrid, Spain; 14 Service Assistance Programs, Regional Health Management Valladolid, Valladolid, Spain; 15 Marquesado Health Centre, Servicio Andaluz de Salud, Granada, Spain; 16 Casablanca Health Centre, Aragonese Institute of Health Sciences, IIS Aragón, Zaragoza, Spain; 17 Department of Medicine and Psychiatry, University of Zaragoza, Zaragoza, Spain; 18 San José Health Centre Linares, Jaén, Spain

**Keywords:** Depression, Genomics

## Abstract

Depression is strongly associated with obesity among other chronic physical diseases. The latest mega- and meta-analysis of genome-wide association studies have identified multiple risk loci robustly associated with depression. In this study, we aimed to investigate whether a genetic-risk score (GRS) combining multiple depression risk single nucleotide polymorphisms (SNPs) might have utility in the prediction of this disorder in individuals with obesity. A total of 30 depression-associated SNPs were included in a GRS to predict the risk of depression in a large case-control sample from the Spanish PredictD-CCRT study, a national multicentre, randomized controlled trial, which included 104 cases of depression and 1546 controls. An unweighted GRS was calculated as a summation of the number of risk alleles for depression and incorporated into several logistic regression models with depression status as the main outcome. Constructed models were trained and evaluated in the whole recruited sample. Non-genetic-risk factors were combined with the GRS in several ways across the five predictive models in order to improve predictive ability. An enrichment functional analysis was finally conducted with the aim of providing a general understanding of the biological pathways mapped by analyzed SNPs. We found that an unweighted GRS based on 30 risk loci was significantly associated with a higher risk of depression. Although the GRS itself explained a small amount of variance of depression, we found a significant improvement in the prediction of depression after including some non-genetic-risk factors into the models. The highest predictive ability for depression was achieved when the model included an interaction term between the GRS and the body mass index (BMI), apart from the inclusion of classical demographic information as marginal terms (AUC = 0.71, 95% CI = [0.65, 0.76]). Functional analyses on the 30 SNPs composing the GRS revealed an over-representation of the mapped genes in signaling pathways involved in processes such as extracellular remodeling, proinflammatory regulatory mechanisms, and circadian rhythm alterations. Although the GRS on its own explained a small amount of variance of depression, a significant novel feature of this study is that including non-genetic-risk factors such as BMI together with a GRS came close to the conventional threshold for clinical utility used in ROC analysis and improves the prediction of depression. In this study, the highest predictive ability was achieved by the model combining the GRS and the BMI under an interaction term. Particularly, BMI was identified as a trigger-like risk factor for depression acting in a concerted way with the GRS component. This is an interesting finding since it suggests the existence of a risk overlap between both diseases, and the need for individual depression genetics-risk evaluation in subjects with obesity. This research has therefore potential clinical implications and set the basis for future research directions in exploring the link between depression and obesity-associated disorders. While it is likely that future genome-wide studies with large samples will detect novel genetic variants associated with depression, it seems clear that a combination of genetics and non-genetic information (such is the case of obesity status and other depression comorbidities) will still be needed for the optimization prediction of depression in high-susceptibility individuals.

## Background

Depression and obesity are common conditions that tend to co-exist. Depression is the most common psychiatric disorder with more than 300 million people suffering from it. At the same time, the prevalence of obesity is a serious worldwide issue and one of the major health challenges of the 21st century [1]. The co-occurrence of both conditions has been designated as one of the most important contributors to the worldwide disability burden, further leading to major personal and public health implications as well as generating an enormous economic and social cost [2, 3]. Given the high prevalence of both disorders and their consequences, understanding the nature of their relationship is a pressing clinical problem.

There is evidence that people with depression are more likely to be obese compared to psychiatrically-healthy controls [4]. Conversely, people with obesity are also more prone to develop depression than normal-weight subjects so that the association between both conditions is bidirectional [5]. Several longitudinal meta-analyses have robustly evidenced this phenomenon, showing how obesity longitudinally increases the risk of developing depression, and vice versa [6–9]. The mechanisms underlying this association remain unclear nonetheless [6]. Behavioral, sociocultural, psychological, and biological factors have been proposed as plausible explanation [3]. Regarding behavioral and sociocultural factors, obesity would lead to depression due to stigma, interpersonal distress, and changes in body image; while depression would lead to obesity as a result of physical inactivity, alcohol abuse, and emotional eating [1, 6, 10, 11]. Interestingly, several biological dysregulations have been further described to derive from such behavioral alterations in both depression and obesity [3]. On the other hand, it might happen that depression and obesity share some molecular disturbances, strongly connected by alterations in the systems involved in homeostatic adjustments and the brain circuitries that integrate mood regulatory responses (e.g., the hypothalamic–pituitary–adrenal (HPA) axis, immuno-inflammatory activation, neuroendocrine regulators of energy metabolism, or the microbiome) [5, 6, 12–14].

Family-based and twin studies have proven a strong heritable component for both depression and obesity, with heritability estimates of ~35% and ~40% for depression and body mass index (BMI) respectively [15–17]. Although in both cases rare genetic variants and other chromosomal aberrations represent the bulk of the genetic load, genome-wide association studies (GWAS) have also identified a great number of associated single nucleotide polymorphisms (SNPs). These SNPs only represent a small fraction of the genetic susceptibility to these diseases nonetheless. While GWAS studies on BMI already identified hundreds of associated SNPs more than a decade ago [18–21], GWAS performed on depression have had notable difficulties for identifying associated variants [22]. Indeed, it has not been until quite recently that two depression GWAS meta-analyses identified 44 [23] and 102 [24] independent and significant loci associated with the disorder. Besides each individual genetic characterization, a shared genetic susceptibility profile has also been revealed for both conditions, which could be another influencing factor for the bidirectional depression–obesity relationship. Particularly, it has been estimated that up to 12% of the genetic component for depression could be shared with obesity [3, 25]. This promising finding has led to innovative approaches aiming to unveil the molecular mechanisms underlying the depression–obesity relationship [26–29].

Although initial expectations for GWASs on depression were high, mentioned SNPs individually account for only small proportions of reported heritability. Consequently, the practice of utilizing individual SNPs to predict depression is now considered a limited approach and other innovative perspectives have emerged to take advantage of available GWAS insights [30]. On this matter, several genomic studies have proposed to study multiple common SNPs collectively to improve the estimation of disease predisposition [31]. Based on the construction of genetic-risk scores (GRSs), that include multiple genetic variants at the same time, these approaches have recently gathered considerable interest [32], and have proven utility identifying groups of individuals who could benefit from the knowledge of their probabilistic susceptibility to disease. In brief, a GRS is usually calculated as a sum of the number of risk alleles carried by an individual, where the risk alleles are defined by the SNPs and their measured effects as detected by GWAS in a particular trait [33]. Although some authors have previously evaluated the performance of GRSs to discriminate depression [23], no study to date has investigated the utility of GRSs for depression prediction in people with obesity accounting for BMI information. On this matter, and given the strong relationship between obesity and depression, it could be possible that the inclusion of BMI information into the model (along with the GRS) elicits an improvement in predictive ability. Previous results from our group have already proved the hypothesis but in the opposite direction; a GRS for obesity improved its performance when the model included information about the depression status of each patient [34].

Therefore, in the present study we aimed: (i) To investigate whether a GRS combining a number of well-defined SNPs associated with depression might have utility for depression prediction in individuals with obesity, (ii) To evaluate whether the predictive ability of the model improves when obesity information is considered as a covariate, and (iii) To obtain a general picture of the cellular and molecular pathways mapped by those SNPs included into the GRS.

## Methods

### Study population

The sample consisted of 2123 community-based individuals (136 depression cases, 1987 controls) from the PredictD-CCRT study; a Cluster, Controlled, Randomized Trial (CCRT). The PredictD-CCRT study was a national, multicentre randomized controlled trial, which had two parallel groups: cluster assignment by primary care center, and a follow-up of 18 months. The aim of this study was to assess the performance of a preventive intervention on the depression incidence, taking into account the level and profile of risk of depression of each individual. The PredictD-CCRT was conducted in 70 primary care centers from 7 Spanish cities. The Spanish National Health Service covers over 95% of the population, providing free medical service, which ensured a representative sample from the south of Spain. Participants were assessed for clinical, psychological, sociodemographic, anthropometric, lifestyle, and other environmental variables. Individuals who also agreed to participate in genetic studies gave specific informed consent and provided a biological sample. This study was approved in each participating city by the corresponding ethics committee, and it was conducted in compliance with the Helsinki Declaration. The PredictD protocol, effectiveness, and cost-effectiveness analyses are fully described and available elsewhere [35–38]. Briefly, patients belonging to the recruiting centers were selected using a systematic random sampling, each 4–6 patients, from the family physician’s appointment lists at random starting points for each day. Family physicians further checked whether the selected patients met any of the following exclusion criteria: age under 18 or over 75 years; inability to understand or speak Spanish; severe mental disorder (psychosis, bipolar, personality disorder…); cognitive impairment; terminal illness; the patient is scheduled to be out of the city more than four months during the 18 months of the follow-up; and persons who attend the primary care center on behalf of the person that initially has the appointment [35].

### Characterization of depression

The psychiatric interview section was conducted by trained interviewers, independently from physicians. These research assistants completed a 20-h training by accredited instructors, in order to guarantee standardization. The section depression of the Composite International Diagnostic Interview (CIDI) was used for the assessment of depression. The CIDI [39, 40] which is a structured interview was used for the diagnosis of depression according to the DSM-IV criteria.

### Characterization of BMI and obesity

Height and weight data from each individual were used to calculate body mass index (BMI) using the formula: weight in kilograms divided by height in square meters (kg/m^2^). International cut-off reference points were applied for obesity categorization (BMI < 25: normal weight, BMI ≥ 25: overweight, and BMI > 30: obesity). Underweight individuals (BMI ≤ 18.5) were excluded from analyses.

### SNP selection

An extensive review of the literature was performed by the research team. Medline and Scopus databases were explored using relevant terms in the field of depression-associated genes (e.g., “depressive disorder”, “major depressive disorder”, “major depression”, “MDD”, “candidate gene”, “SNP”, “polymorphism”, “loci”). SNPs were initially selected based on two criteria: (i) SNPs from candidate genes reported in case-control studies on depression and replicated in more than one independent study, or in loci having a significant potential role in depression (i.e., loci involved in well-established pathways associated with depression: the hypothalamic–pituitary–adrenal (HPA) axis [41] and the serotonergic system [42]) (*n* = 25); (ii) SNPs associated with depression from GWAS or meta-analyses establishing a *P*-value cut-off of *P* ≤ 7 × 10^−6^ (*n* = 47). The information obtained from each approach was then combined and compared to the list of SNPs available from Illumina technology (San Diego, California), so that a definitive list of candidate variants was obtained: 6 and 10 SNPs initially selected from candidate gene studies and GWAS, respectively, were discarded in this step. Finally, 56 SNPs were selected for downstream analyses: (i) 19 SNPs from candidate gene association studies [43–55] and (ii) 37 SNPs associated with depression in GWAS or meta-analyses [56–68]. Detailed information for the final 56 candidate SNPs included in the analyses and their mapped loci is available in Supplementary Table 1a, b.

We further investigated functional and biological databases for additional information about identified top genetic regions on depression. Among the genes mapped by the selected SNPs, there are genes associated with depression-related pathways [25, 69–74]. A pathway enrichment analysis (PEA) of those genes was performed with the gprofiler2 R package [75]. Pathways that were significantly over-represented in the gene list, as compared to all known genes in the genome, were obtained from Reactome and WikiPathways databases (Supplementary Fig. 1). The *P*-value of the enrichment of the different pathways was computed using a Fisher’s exact test and multiple-test corrections were applied, considering false discovery rates (FDRs), that measures expected proportion of false significant matches within results. Details for the enrichment analysis can be found in Supplementary Table 2. Besides, following a recent detailed protocol [76], statistically significant pathways (FDR-adj.*P* < 0.05) were used to build an enrichment map in the ‘EnrichmentMap’ Cytoscape app [77] with the aim to collapse redundant pathways into a single biological theme rather than including general and specific pathways with many shared genes (Supplementary Fig. 2).

### Genotyping

A saliva sample was obtained from each participant using the Oragene DNA saliva collection kit (OG-500; DNA Genotek Inc.). DNA extraction was performed using standard procedures. DNA concentration was measured by absorbance measure using the Infinite® M200 PRO multimode reader (Tecan, Research Triangle Park, NC).

**Fig. 1:**
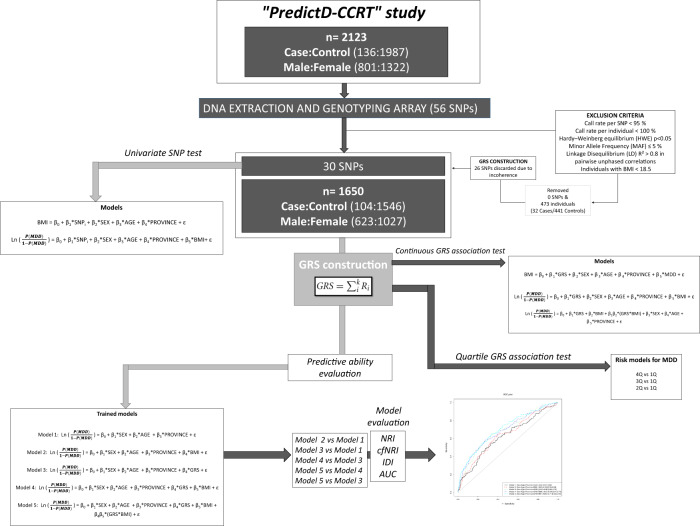
Complete workflow detailing the study design and statistical analyses performed: quality control process, association analysis and construction and evaluation of predictive models. AUC area under the receiver operating characteristic curve, cfNRI the category-free net reclassification improvement, HWE Hardy–Weinberg equilibrium, IDI integrated discrimination improvement, LD linkage disequilibrium, MAF minor allele frequency, MDD major depressive disorder, NRI net reclassification improvement, SNP single nucleotide polymorphism.

Genotyping was performed using the TaqMan® OpenArray^TM^ Genotyping System (Applied Biosystems, Foster City, CA) following the manufacturer’s instructions. Raw data were analyzed with the TaqManGenotyper v1.2 software (Thermo Fisher Scientific). SNPs showing a linkage disequilibrium (LD) value of *R*² > 0.8 in pairwise unphased correlations were removed from the selection. For all candidate markers, we further evaluated call rate, Hardy–Weinberg equilibrium (HWE), and minor allele frequency (MAF). MAFs of all SNPs were ≥0.05 and similar to those reported for Iberian populations in Spain in phase 3 of the 1000 Genomes Project. To account for the presence of genotyping errors, all SNPs with less than a 95% call rate were excluded from the analyses. In relation to HWE, the Wigginton’s exact test was applied only in controls at an alpha level of 0.05. After all quality control checks, the selected 56 markers were available for downstream analyses. A complete workflow detailing the whole SNP selection procedure can be found in Fig. 1.

### Genetic-risk score construction (GRS)

To explore whether common variants with small risk effects on depression predict depression occurrence in our sample, a GRS was constructed following an unweighted approach, as implemented in the *PredictAbel* R package [78]. In this approach, a GRS is calculated for each individual based on the number of risk alleles for depression, without accounting for each SNP effect size. The motivation underlying this choice is the fact that most of the selected markers came from individual genetic studies, where no robust beta values were available. All initially selected SNPs from the literature (56 SNPs, as can be seen in Supplementary Table 1a), were assessed for association in our sample applying univariate regression analyses on depression. Although most of these SNPs were not significantly associated with depression in our sample, all effect sizes and direction of associations were compared to those reported in the literature for each selected SNP. With the aim of constructing a robust genetic-risk score, we only selected those SNPs showing concordant associations (in terms of effect size direction) between the association signal reported in the literature and the association signal reported in our sample. As a result, 30 SNPs from the 56 markers were selected for the construction of the GRS. The rationale behind this procedure is the fact that many of the selected SNPs might not be significant in our population while still being associated with depression according to other studies. For these SNPs that individually do not show statistical association, constructing a GRS is of special interest since we can combine their small effects so joined, they report a significant relationship with depression. SNPs in which the minor allele was reported as a protective marker (instead of a risk variant) were flipped in order to compute the risk score. Since the GRS cannot be estimated if any of the markers present a non-callable genotype for a certain individual, subjects showing a call rate <100% for any of the 30 candidate SNPs were removed from the analyses. As a result, 1650 individuals (104 depression cases and 1546 controls) were considered for the GRS construction. Details for the SNPs finally included in the GRS can be found in Supplementary Table 1a.

### Statistical analysis

A complete workflow detailing the study design and statistical analyses performed can be found in Fig. 1. Differences between cases and controls for main clinical characteristics were analyzed using the Student’s *t* test, the Welch’s test, or the U Mann–Whitney test for quantitative variables. The Pearson’s Chi-squared test was used for investigating group differences in categorical variables instead. Cohen’s *d* and Cramér’s *V* were calculated to assess effect sizes in quantitative and qualitative variables, respectively.

Binary logistic regression models were employed to test the effect of each individual SNP on depression under an additive genetic model of inheritance (thereafter named in the paper as univariate SNP analyses). We performed a post hoc power analysis based on a Z test for logistic regression using G-Power software. Power estimation was conceived for a logistic regression model including the GRS and the rest of adjusting covariates. Under the assumption of a normal distribution for the GRS in our sample, the power of our logistic regression in the *N* = 1650 sample, was estimated 99.99% (e.g., there is a 99.99% chance of correctly rejecting the null hypothesis that a particular value of the GRS is not associated with the value of the outcome variable (depression), with 1650 participants). Multiple linear regressions were used for the univariate SNP analyses on BMI. Regression models were evaluated by model control investigating linearity of effects on outcome(s), consistency with a normal distribution, and variance homogeneity. Continuous variables were tested for normality using the Shapiro–Wilk test and transformed when necessary by means of the natural log or the rank-based inverse normal transformation. All regression models employed are detailed in Fig. 1. Given the number of genetic markers analyzed, we considered false discovery rates (FDRs) calculated as in Benjamini and Hochberg to correct for multiple-hypothesis testing in univariate SNP analyses [79]. Regarding GRS analyses, logistic regression models were applied to test whether higher genetic-risk scores were observed for depression cases than controls. Logistic regression models were also applied for comparing participants presenting a high-risk genetic profile (Q2, Q3, or Q4) *vs*. those belonging to the reference quartile (Q1). Multiple linear regression was employed to investigate association between continuous GRS and BMI. Model deviance of logistic regressions (D²) was calculated to assess the amount of outcome variability explained by each group of variables. All tested models in our work were properly adjusted by confounders such as sex, age, province (geographical location), or BMI whenever necessary (Fig. 1).

To assess the predictive ability of the constructed GRS, five different predictive models were trained and evaluated in our sample (see trained models in Fig. 1). The area under the receiver operating characteristic (ROC) curve (AUC) was calculated for each model and all possible comparisons between constructed models were tested for statistical significance in terms of prediction improvement. Besides AUC, three recently proposed statistical metrics were also adopted to quantify the added predictive value of each model with respect to its immediate prior. These statistical metrics were the integrated discrimination improvement (IDI), the net reclassification improvement (NRI), and the category-free NRI (cfNRI). All of them have been previously described [80]. Since no established risk categories exist in depression, the NRI was applied according to standard risk categories (low (<5%), medium (5% to <25%), or high (≥25%)) [80]. For this reason, the cfNRI and the IDI were preferred estimators than NRI in our study. Discrimination plots, predictiveness curves, prior posterior risk curves, and risk distribution plots were obtained for each trained model (data not shown but available upon request). All predictive assessments were conducted using the *PredictABEL* and the *pROC* R packages [78, 81]. All statistical analyses were performed in R environment, version 3.4.5 (R Project for Statistical Computing).

Besides constructing our models in the whole population (interpretable models), we also implemented a sampling validation procedure. Particularly, K-fold cross-validation is a great method in case the classes are not equally balanced in a dataset. The use of this sort of validation consists of dividing the dataset in k groups or folds of samples (of equal sizes, if possible). Thus, the learning process is done with k−1 folds (training), and the evaluation of model’s performance is done with the fold left out (testing). This iterative method helps to create models using different folds, and evaluate the model’s performance through different metrics (with the fold left out). In Particular, we opted for a 5-fold cross-validation. To create the folds we used the function createFolds() from the caret package. To calculate the rest of the metrics we used the reclassification() function from the predictABLE package.

## Results

### Demographic characteristics

A complete workflow detailing the adopted study design and the statistical analysis conducted can be found in Fig. 1. General characteristics of the study population by experimental condition are summarized in Supplementary Table 3. After excluding subjects with any missing genotypes or BMI under 18.5, a total of 1650 participants were finally included in our analysis (104 depression cases and 1546 controls). A significantly higher BMI was found in depression cases than in control (*P* = 0.02; Cohen’s *d* = 0.24). Furthermore, we found statistically significant differences in the case-control proportion between the different provinces of recruitment (*P* = 0.008; Cramér’s *V* = 0.103), suggesting that the geographical location of participants could be an important confounding variable to adjust genetics models for. The mean age of the participants was slightly higher in the group with depression than in controls, although this difference was not statistically significant (*P* = 0.19; Cohen’s *d* = 0.13). Regarding sex, 62.24% of the total sample were females with no sex differences observed between cases and controls (*P* = 0.64; Cramér’s *V* = 0.12).

### Genetic association analyses on depression

Five SNPs from the 56 candidate genetic variants showed a significant association with depression status in the univariate analyses (Supplementary Table 4). While the reference minor alleles of SNPs rs6537837 and rs242939 (mapping the *GNAI3* and *CRHR1* genes, respectively) were reported as protective markers for depression, the reference minor alleles for the rs349475, rs310501, and rs1800532 (mapping the genes *LINC02223;CDH18*, *VCAN;HAPLN1*, and *TPH1*) were identified as risk markers for depression. Univariate SNP analyses for depression were adjusted for all pertinent confounders as illustrated in Fig. 1. Although none of these results remained statistically significant after strict multiple-hypothesis correction by FDR (alpha = 0.05), all associations were nominally confirmed using Fisher’s exact tests. The genetic location of each SNP (i.e., exonic, intronic, intergenic, upstream, or downstream) is shown in Supplementary Table 1a. Surprisingly, all significant SNPs were identified as intronic or intergenic variants.

**Fig. 2:**
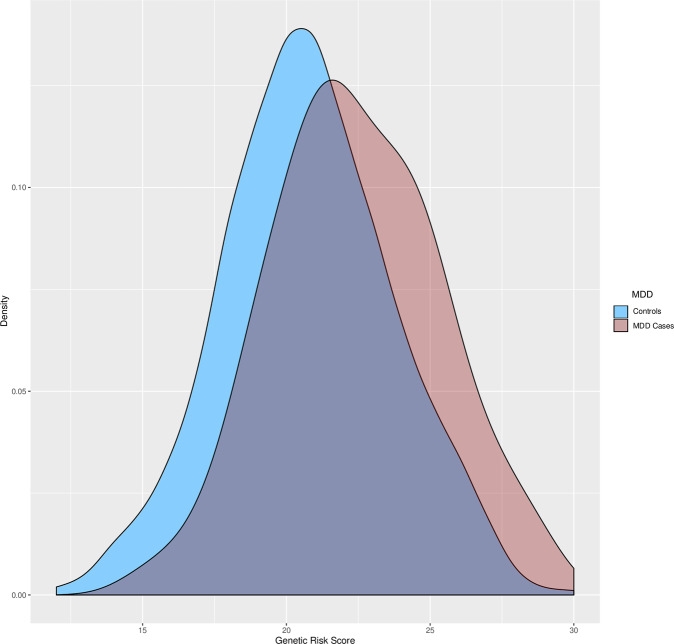
Density distribution plot of the constructed GRS in our population. MDD major depressive disorder.

**Fig. 3:**
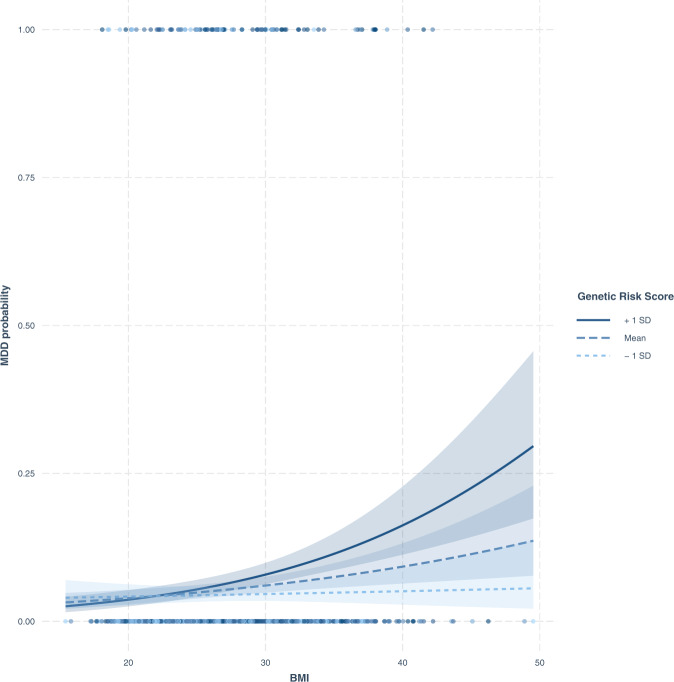
Graphical representation of the direction and magnitude of the GRS*BMI interaction. BMI body mass index, MDD major depressive disorder, SD standard deviation.

From these analyses, 30 SNPs were carefully selected (as they showed similar directional effect compared to the literature findings) and incorporated into an unweighted GRS. The GRS was tested for association with depression status as described in the “Methods” section and in Fig. 1. The density distribution plot of the constructed GRS in our population is presented in Fig. 2. The mean (and standard deviation) of the GRS in the whole sample was 20.82 (2.97), being 22.38 (2.91) in depression cases and 20.71 (2.94) in controls, being this difference statistically significant (*P* = 1.02 × 10^−7^; Cohen’s *d* = 0.57) (Supplementary Table 3). Interestingly, a logistic regression model adjusted for sex, age, province, and BMI revealed a strong risk association between the GRS and the depression status, so that the odds of being depressed were estimated to increase by factor 1.35 for each additional risk allele in the GRS (OR = 1.35; CI 95% = [1.13, 1.27]; *P* = 1.35 × 10^−7^). The depression variability attributable to the genetic component in the model was estimated at 4.17%. When comparing individuals presenting the highest risk scores (Q4) to those belonging to the first quartile (Q1) a stronger association was evidenced (OR = 4.19; CI 95% = [2.3, 7.62]; *P* = 2.76 × 10^−6^). When comparing individuals in the third quartile (Q3) to those belonging to the first quartile (Q1), the association was quantified with an OR = 2.37 (CI 95% = [1.25, 4.46]; *P* = 0.008). The remaining comparison (Q2-*vs*-Q1) reported a non-significant result otherwise (OR = 1.73; CI 95% = [0.92, 3.26]; *P* = 0.08). When modeling BMI on depression in a model adjusted for sex, age and province, no significant association was reported (OR = 1.2; CI 95% = [0.98, 1.46]; *P* = 0.09). On the other hand, the most intriguing result was the interaction found between the GRS and BMI (further adjusted for age, sex, and province), with depression (OR = 1.14; CI 95% = [1.07–1.20]; *P* = 1 × 10^−4^). The direction and magnitude of this interaction can be observed in Fig. 3, and suggest the existence of gene-environment interaction phenomena by which BMI increases the genetics-conferred risk of depression in high-susceptibility individuals.

### Prediction of depression

**Fig. 4:**
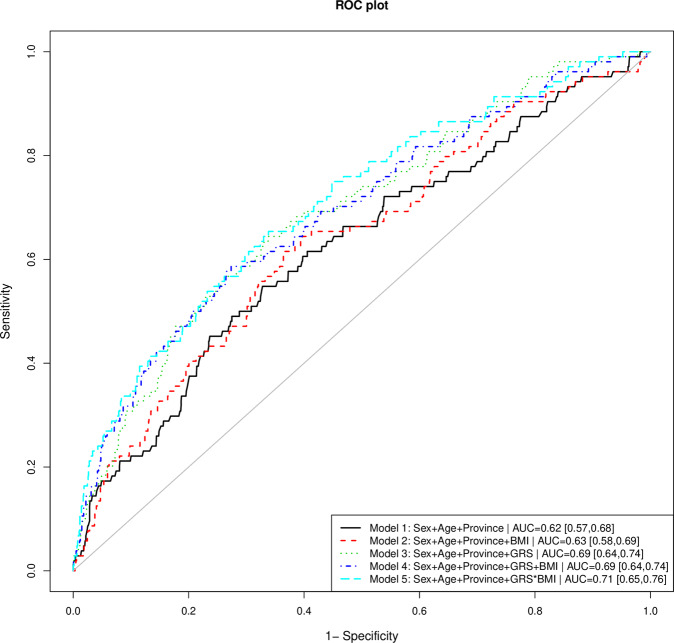
Evaluation of the predictive ability of the constructed predictive model using AUC. AUC area under the receiver operating characteristic curve, GRS genetic-risk score.

To demonstrate the validity of the GRS for the prediction of depression, five logistic regression models were constructed, trained, and evaluated in our sample. These models comprised a model with classical demographic information only (age, sex, and province), named model 1, and other four additional models that further included (alone or combined) BMI or GRS information (see Fig. 1 for more details). The predictive ability of each model was evaluated using AUC. Statistical significance and magnitude of improvements in predictions between models were further assessed by means of the metrics NRI, cfNRI, and IDI, as described in the “Methods” section. Results for all models and performed comparisons are presented in Fig. 4 and Table 1. The lowest predictive ability corresponded to the model 1, incorporating classical demographic information only (AUC = 0.62, 95% CI = [0.57, 0.68]) (Fig. 4). The inclusion of the GRS into this model (model 3) reported an increase in the AUC up to 0.69 (95% CI = [0.64, 0.74]) and (*P* = 1 × 10^−5^ for cfNRI and IDI). Instead, the inclusion of BMI into the classical demographic model (model 2) did not provoke any improvement in the AUC of model 1 (Table 1). A model combining both the GRS and BMI as marginal terms alongside the classical demographic variables (model 4) barely improved the AUC reported for the model 3 (with the GRS as a marginal term). Surprisingly, a model incorporating an interaction term between the GRS and the BMI (model 5) achieved the higher predictive ability for depression (AUC = 0.71, 95% CI = [0.65, 0.76]). The significance of this improvement of model 5 with regard to both model 3 and 4 was estimated at *P* = 0.009 for IDI and NRI. Presented results correspond to the model constructed in the whole sample. In addition, we cross-validated our findings employing a 5-fold CV procedure. Our main conclusions remained, although the AUC of all models slightly decreased (Supplementary Tables 5 and 6). The results shown in these tables correspond to the average metrics across folds (k = 5).

**Table 1:** Statistics for model improvement with the addition of genetic and non-genetic-risk factors for MDD.

	Initial model: Model 1 Final model: Model 2	Initial model: Model 1 Final model: Model 3	Initial model: Model 3 Final model: Model 4	Initial model: Model 3 Final model: Model 5	Initial model: Model 4 Final model: Model 5
NRI	−0.03 (−0.08,0.009)	0.09 (−0.001,0.17)	−6e−04 (−0.04,0.04)	0.11 (0.02,0.19)	0.11 (0.02,0.19)
NRI *P-*value	0.12	0.05	0.98	**0.01**	**0.009**
cfNRI	0.19 (−0.006,0.39)	0.43 (0.24,0.63)	0.16 (−0.04,0.36)	0.24 (0.04,0.43)	0.17 (-0.03,0.37)
cfNRI *P-*value	0.06	**1e**−**05**	0.11	**0.01**	0.09
IDI	0.002 (−6e−04,0.005)	0.02 (0.01,0.03)	0.003 (5e−04,0.006)	0.02 (0.007,0.03)	0.01 (0.005,0.02)
IDI *P-*value	0.12	**1e**−**05**	**0.02**	**0.001**	**0.001**

Model 1 (Sex+Age+Province), Model 2 (Sex+Age+Province+BMI), Model 3 (Sex+Age+Province+GRS), Model 4 (Sex+Age+Province+GRS + BMI), and Model 5 (Sex+Age+Province+GRS*BMI). The 95% confidence intervals are shown in parentheses. Statistically significant results are highlighted in bold.

*NRI* net reclassification improvement, *cfNRI* category-free NRI, *IDI* integrated discrimination improvement, *AUC* area under the curve of the receiver operator characteristic curve.

### Genetic pleiotropy on BMI

Given previous evidence of a shared genetic-risk profile between depression and BMI, we investigated whether an association could exist between candidate SNPs and BMI in our sample. For that purpose, both univariate SNP and GRS-based analyses were performed with BMI as outcome variable (see Fig. 1 for more details regarding adjusting covariates). As a result, no significant association was reported between the GRS and BMI (OR = 1.2, 95% CI = [0.98−1.46], and *P* = 0.09) (Supplementary Fig. 3). In univariate SNP analyses, only the rs12457996 (mapping the *SYT4* gene) showed a significant association (Supplementary Table 7) being the C allele associated with a lower BMI in our sample (*P* = 0.03). The association did not remain statistically significant after multiple-testing correction.

## Discussion

In this study, we constructed an unweighted GRS, including 30 depression-associated genetic-risk variants from previous GWAS and candidate gene studies on depression (Fig. 1) [57–63]. As a first goal, we aimed to investigate whether the constructed GRS was associated with depression as well as if it was able to predict depression with enough precision and accuracy. Given the strong connection between depression and obesity, we also aimed to elucidate whether the predictive ability of the GRS improved with the inclusion of BMI information for each individual. As a result, we found that the GRS is strongly associated with depression status and that it presents a not negligible depression predictive ability by itself. Remarkably, we showed how the interaction of BMI information with the GRS improves the predictive ability of the genetic component, deriving to a predictive ability of certain clinical relevance (AUC = 0.71). This result goes in line with recent approaches in which a close relationship between both conditions has been described [26–28] and complements our previous study in which we demonstrated the opposite relation [34].

We found that higher scores from the constructed GRS are strongly associated with a greater prevalence of depression in our sample (*P* = 1.35 × 10^−7^) (Fig. 2). In these analyses, the genetic component represented by the GRS accounted for 4.17% of the depression heritability. For the accomplishment of all these analyses, an unweighted GRS approach was employed, instead of a weighted GRS, due to the lack of GWAS or genetic meta-analyses providing robust estimates for the effects of the SNPs of interest in the literature [56–67]. Despite not using a weighted approach, our unweighted GRS demonstrated a strong association with depression, which is in line with previous reports on the clinical utility of GRS unweighted approaches [34, 82, 83].

The GRS showed a stronger association with depression than individual SNPs. Thus, it is quite probable that common genetic variants tested here represent only a small and cumulative contribution to the whole genetic susceptibility profile of depression [23, 24, 84], which is a commonly observed phenomenon in the genetic architecture of many complex diseases [85]. In our study, the finding of SNPs eliciting small and cumulative risk effects on depression was further supported by the fact that the greater and more significant differences were observed for the comparisons between individuals presenting a considerable number of risk variants (Q4) and individuals from the bottom reference quartile (Q1), which are individuals barely presenting risk alleles (OR = 4.19; CI 95% = [2.3, 7.62]; *P* = 2.76 × 10^−6^).

Both alone and in combination with classic demographic information, the GRS has demonstrated a good performance for the prediction of depression status in our sample. Contrary to the GRS, the addition of BMI information alone to the basic model did not show an improvement of its performance (although an increase in the AUC was reported, it did not reach statistical significance). The further inclusion of BMI information as an interaction term (along with the GRS) elicited a significant improvement in the clinical prediction of depression (*P*-value IDI = 0.001 and *P*-value NRI = 0.009) (Table 1). Particularly, BMI was identified as a trigger-like risk factor for depression acting in a concerted way with the GRS component (Fig. 3). These findings, therefore, support the existence of a link between obesity and depression and reinforce the theory that the relationship is bidirectional.

In order to obtain a general picture of the cellular and molecular pathways mapped by analyzed SNPs, an enrichment functional analysis was conducted. Observed gene overlapping between some of the significant pathways suggests that those cellular mechanisms in which are involved could provide part of the disease map in depression. Associations between those obtained biological pathways and depression have been found in previous studies [25, 69, 70]. In this regard, for example, there is evidence that confirms the antidepressant potential of ECM remodeling after chronic stress by mean of intra-cortical degradation of perineuronal nets (PNNs) [71]; or the role of the PI3K-Akt signaling pathway in proinflammatory regulatory mechanisms, given that neuron inflammation and inflammatory cytokine production contribute to the pathology of depression [72]; as well as, the alteration of circadian rhythms and disturbances of sleep [69, 73]. In this way, such evidence not only gives support to the use of certain polymorphisms as a predictive tool, but also helps us to contextualize which mechanisms are being altered and to have a more functional perspective of this analysis.

There are certainly some limitations that should be mentioned. Some of the main drawbacks from this study include a high unbalanced design between depression cases and control as well as the absence of analyzed SNPs from the recently published meta-analysis list [23, 24]. Therefore, generated hypotheses here would require more detailed characterization in bigger and independent cohorts.

In summary, we found that a GRS based on 30 depression-associated risk loci was significantly associated with depression. Although GRS on its own explained only a small amount of variance of depression, a significant novel feature of this study is that including non-genetic-risk factors such as BMI together with a GRS came close to the conventional threshold for clinical utility used in ROC analysis and improves the prediction of depression. This has potential clinical implications as well as implications for future research directions in exploring the links between depression and obesity-associated disorders. While it is likely that future genome-wide studies with very large samples will detect variants other than the common ones, it seems probable that a combination of non-genetic information will still be needed to optimize the prediction of obesity.

## Supplementary information

Supplementary legends

Supplementary Table 1a

Supplementary Table 1b

Supplementary Table 2

Supplementary Table 3

Supplementary Table 4

Supplementary Table 5

Supplementary Table 6

Supplementary Table 7

Supplementary Figure 1

Supplementary Figure 2

Supplementary Figure 3
