# Supplementary material for: Body mass index interacts with a genetic-risk score for depression increasing the risk of the disease in high-susceptibility individuals
Source: Transl Psychiatry. 2022 Jan 24;12:30. doi: 10.1038/s41398-022-01783-7 (PMC8786870; doi:10.1038/s41398-022-01783-7)
Supplement: Supplementary file 1 — Supplementary legends [file 41398_2022_1783_MOESM1_ESM.docx]

**Supplementary legends:**

Table S1a. Genomic information for all analyzed SNPs and loci. SNP ids in bold were finally included in the genetic risk score after quality control pruning. Abbreviations: SNP, Single Nucleotide Polymorphism; CHR, Chromosome; REF, Reference minor allele; ALT, Alternative allele.

Table S1b. Candidate genes and number of genes analyzed per selection category.

Table S2. Reported significant pathways with P values adjusted for multiple testing correction (FDR-adj.P<0.05) from Reactome and WikiPathways databases. Abbreviations: REAC, Reactome; WP, WikiPathways.

Table S3. Demographic characteristics of the study population by experimental condition. Abbreviations: BMI, body mass index; GRS, genetic risk score. Data are expressed as mean (standard deviation). p-values of the categorical variables Sex and Province were obtained after performing a χ² test.

Table S4. SNPs-based association test on MDD status. These results come from a single logistic regression model with all SNPs as covariates. Abbreviations: SNP, Single Nucleotide Polymorphism; OR, odds ratio; SE, standard error; ci.lo, 95 % lower confidence interval; ci.up, 95 % upper confidence interval and FDR, False Discovery Rate.

Table S5. Average statistics for model improvement with addition of genetic and non-genetic risk factors for MDD after implementation of 5-fold cross-validation procedure. Model 1 (Sex+Age+Province); Model 2 (Sex+Age+Province+BMI); Model 3 (Sex+Age+Province+GRS), Model 4 (Sex+Age+Province+GRS+BMI) and Model 5 (Sex+Age+Province+GRS*BMI). Abbreviations: NRI, net reclassification improvement; cfNRI, category-free NRI; IDI, integrated discrimination improvement. The 95% confidence intervals are shown in parentheses.

Table S6. Average AUC obtained for each model in the test sample after the implementation of 5-fold cross-validation procedure. Model 1 (Sex+Age+Province); Model 2 (Sex+Age+Province+BMI); Model 3 (Sex+Age+Province+GRS), Model 4 (Sex+Age+Province+GRS+BMI) and Model 5 (Sex+Age+Province+GRS*BMI). Abbreviations: AUC, area under the curve of the receiver operator characteristic curve.

Table S7. SNP-based association tests on BMI. Abbreviations: SNP, Single Nucleotide Polymorphism; βeta, Beta effect obtained under an additive genetic model of inheritance; SE, standard error, ci.lo, 95 % lower confidence interval; ci.up, 95 % upper confidence interval and FDR, false discovery rate.

Figure-S1.tiff. Figure S1. Dotplot showing the significantly over-represented pathways (FDR-adj.P<0.05) from Reactome and WikiPathways databases in the genes mapped by the SNPs included in the genetic risk score.

Figure-S2.tiff. Figure S2. Enrichment map of the pathway enrichment analysis in which each node represents one pathway and node colors correspond to the degree of significance. Edge thickness indicates the amount of genes shared by two gene sets and node sizes are proportional to the number of genes assigned to the corresponding pathway term.

Figure-S3.tiff. Figure S3. Histogram of Genetic Risk Score in relation to BMI.
