# Supplementary material for: Body mass index interacts with a genetic-risk score for depression increasing the risk of the disease in high-susceptibility individuals
Source: Transl Psychiatry. 2022 Jan 24;12:30. doi: 10.1038/s41398-022-01783-7 (PMC8786870; doi:10.1038/s41398-022-01783-7)
Supplement: Supplementary file 2 — Supplementary Table 1a [file 41398_2022_1783_MOESM2_ESM.docx]

**Table S1a.** Genomic information for all analyzed SNPs.

| **SNP** | **CHR** | **START** | **END** | **REF** | **ALT** | **TYPE** | **GENE** | **REFERENCE** |
| --- | --- | --- | --- | --- | --- | --- | --- | --- |
| rs1801133 | chr1 | 11856378 | 11856378 | G | A | exonic | MTHFR | 43, 43b, 43c |
| **rs12049330** | chr1 | 110031188 | 110031188 | T | G | intronic | ATXN7L2 | 62 |
| rs6537837 | chr1 | 110119732 | 110119732 | C | T | intronic | GNAI3 | 62 |
| rs606149 | chr1 | 193921548 | 193921548 | C | T | intergenic | LINC01031;NONE | 58 |
| **rs11009175** | chr10 | 33294775 | 33294775 | G | A | intergenic | ITGB1;NRP1 | 65 |
| rs1780436 | chr10 | 34257612 | 34257612 | G | A | intergenic | LINC00838;PARD3 | 57 |
| **rs12415800** | chr10 | 69624180 | 69624180 | G | A | intergenic | DNAJC12;SIRT1 | 67 |
| **rs1800532** | chr11 | 18047816 | 18047816 | G | T | intronic | TPH1 | 44, 44b, 44c |
| rs623580 | chr11 | 18063977 | 18063977 | A | T | intergenic | TPH1;SAAL1 | 45 |
| **rs6265** | chr11 | 27679916 | 27679916 | C | T | exonic | BDNF | 55, 55b |
| rs4238010 | chr12 | 4118317 | 4118317 | G | A | intergenic | PARP11;CCND2-AS1 | 59 |
| **rs5443** | chr12 | 6954875 | 6954875 | C | T | exonic | GNB3 | 46, 46b, 46c |
| **rs9943849** | chr12 | 69370756 | 69370756 | C | T | intergenic | CPM;CPSF6 | 60 |
| rs1545843 | chr12 | 84564068 | 84564068 | G | A | intergenic | TMTC2;SLC6A15 | 61 |
| **rs7326068** | chr13 | 21209512 | 21209512 | G | A | intronic | IFT88 | 56 |
| rs9526236 | chr13 | 47388527 | 47388527 | C | T | intergenic | ESD;HTR2A | 45 |
| rs6311 | chr13 | 47471478 | 47471478 | C | T | upstream | HTR2A | 48, 48b |
| rs1927745 | chr13 | 107780316 | 107780316 | G | A | intergenic | LINC00443;FAM155A | 65 |
| **rs8023445** | chr15 | 49192791 | 49192791 | T | C | intronic | SHC4 | 68 |
| **rs12912233** | chr15 | 61267096 | 61267096 | C | T | intronic | RORA | 65 |
| rs9939609 | chr16 | 53820527 | 53820527 | T | A | intronic | FTO | 47, 47b |
| **rs12446956** | chr16 | 73501536 | 73501536 | T | C | intergenic | LINC01568;LOC101928035 | 64 |
| rs10514585 | chr16 | 83284338 | 83284338 | G | A | intronic | CDH13 | 65 |
| rs9646303 | chr16 | 87461969 | 87461969 | G | A | intronic | ZCCHC14 | 66 |
| **rs8070473** | chr17 | 33843512 | 33843512 | T | G | intergenic | SLFN12L;SLFN14 | 65 |
| **rs110402** | chr17 | 43880047 | 43880047 | G | A | intronic | CRHR1;LINC02210-CRHR1 | 45 |
| **rs242924** | chr17 | 43885367 | 43885367 | G | T | intronic | CRHR1;LINC02210-CRHR1 | 45 |
| **rs242939** | chr17 | 43895579 | 43895579 | C | T | intronic | CRHR1;LINC02210-CRHR1 | 45 |
| **rs1876828** | chr17 | 43911525 | 43911525 | C | T | intronic | CRHR1;LINC02210-CRHR1 | 45 |
| **rs12457996** | chr18 | 40872273 | 40872273 | T | C | intergenic | SYT4;LINC01478 | 64 |
| **rs17077540** | chr18 | 65285279 | 65285279 | A | G | ncRNA_intronic | LOC643542 | 62 |
| **rs429358** | chr19 | 45411941 | 45411941 | T | C | exonic | APOE | 49, 49b |
| **rs7412** | chr19 | 45412079 | 45412079 | C | T | exonic | APOE | 49, 49b |
| rs7565124 | chr2 | 20261685 | 20261685 | G | A | intergenic | LAPTM4A;SDC1 | 68 |
| rs1449984 | chr2 | 23414651 | 23414651 | A | G | intergenic | LINC01884;KLHL29 | 65 |
| **rs882632** | chr2 | 29280761 | 29280761 | C | T | intergenic | TOGARAM2;C2orf71 | 62 |
| rs724568 | chr2 | 67942480 | 67942480 | C | A | intergenic | ETAA1;LINC01812 | 63 |
| rs2828520 | chr21 | 25143119 | 25143119 | G | A | intergenic | D21S2088E;LINC01689 | 62 |
| **rs4680** | chr22 | 19951271 | 19951271 | G | A | exonic | COMT | 50, 50b, 50c |
| rs9870680 | chr3 | 7529555 | 7529555 | C | T | intronic | GRM7 | 63 |
| rs10514718 | chr3 | 61413814 | 61413814 | C | G | intergenic | FHIT;PTPRG | 62 |
| **rs2173763** | chr3 | 122329160 | 122329160 | A | G | intronic | PARP15 | 66 |
| rs644695 | chr3 | 177291697 | 177291697 | A | G | ncRNA_intronic | LINC00578 | 63 |
| rs1969253 | chr3 | 183876512 | 183876512 | A | C | intronic | DVL3 | 66 |
| **rs7647854** | chr3 | 184876783 | 184876783 | G | A | intergenic | C3orf70;EHHADH-AS1 | 64 |
| **rs349475** | chr5 | 19440168 | 19440168 | T | C | intergenic | LINC02223;CDH18 | 65 |
| **rs6295** | chr5 | 63258565 | 63258565 | C | G | upstream | HTR1A | 51, 51b |
| **rs10473984** | chr5 | 76267126 | 76267126 | G | T | intergenic | CRHBP;AGGF1 | 52 |
| rs7713917 | chr5 | 78829249 | 78829249 | A | G | intergenic | HOMER1;PAPD4 | 60 |
| **rs310501** | chr5 | 82889910 | 82889910 | G | A | intergenic | VCAN;HAPLN1 | 58 |
| rs6296 | chr6 | 78172260 | 78172260 | C | G | exonic | HTR1B | 53, 53b |
| rs2270007 | chr7 | 30699972 | 30699972 | G | C | intronic | CRHR2 | 54 |
| **rs2715148** | chr7 | 82450035 | 82450035 | A | C | UTR3 | PCLO | 57 |
| rs17864092 | chr7 | 126639604 | 126639604 | T | C | intronic | GRM8 | 65 |
| rs10265216 | chr7 | 130900121 | 130900121 | T | A | intronic | MKLN1 | 63 |
| **rs1106634** | chr8 | 20066049 | 20066049 | G | A | intronic | ATP6V1B2 | 63 |

SNP ids in bold were finally included in the genetic risk score after quality control pruning. Abbreviations: SNP, Single Nucleotide Polymorphism; CHR, Chromosome; REF, Reference minor allele; ALT, Alternative allele.

**References**

[43. Wu Y-L, Ding X-X, Sun Y-H, Yang H-Y, Chen J, Zhao X, et al. Association between MTHFR C677T polymorphism and depression: An updated meta-analysis of 26 studies. Progress in Neuro-Psychopharmacology and Biological Psychiatry. 2013 Oct;46:78–85.](https://www.zotero.org/google-docs/?jPUPOW)

43b. Bjelland, I., Tell, G. S., Vollset, S. E., Refsum, H., & Ueland, P. M. (2003). Folate, vitamin B12, homocysteine, and the MTHFR 677C->T polymorphism in anxiety and depression: the Hordaland Homocysteine Study. Archives of general psychiatry, 60(6), 618–626.

43c. Lewis, S. J., Lawlor, D. A., Davey Smith, G., Araya, R., Timpson, N., Day, I. N., & Ebrahim, S. (2006). The thermolabile variant of MTHFR is associated with depression in the British Women's Heart and Health Study and a meta-analysis. Molecular psychiatry, 11(4), 352–360.

[44. Arias B, Fabbri C, Gressier F, Serretti A, Mitjans M, Gastó C, et al. TPH1, MAOA, serotonin receptor 2A and 2C genes in citalopram response: possible effect in melancholic and psychotic depression. Neuropsychobiology. 2013;67(1):41–7.](https://www.zotero.org/google-docs/?jPUPOW)

44b. Serretti, A., Zanardi, R., Cusin, C., Rossini, D., Lorenzi, C., & Smeraldi, E. (2001). Tryptophan hydroxylase gene associated with paroxetine antidepressant activity. European neuropsychopharmacology : the journal of the European College of Neuropsychopharmacology, 11(5), 375–380.

44c. Andre, K., Kampman, O., Viikki, M., Illi, A., Setälä-Soikkeli, E., Poutanen, O., Mononen, N., Leinonen, E., & Lehtimäki, T. (2013). TPH1 A218C polymorphism and temperament in major depression. BMC psychiatry, 13, 118.

[45. Ching-López A, Cervilla J, Rivera M, Molina E, McKenney K, Ruiz-Perez I, et al. Epidemiological support for genetic variability at hypothalamic-pituitary-adrenal axis and serotonergic system as risk factors for major depression. Neuropsychiatr Dis Treat. 2015;11:2743–54.](https://www.zotero.org/google-docs/?jPUPOW)

[46. Prestes AP, Marques FZC, Hutz MH, Bau CHD. The GNB3 C825T polymorphism and depression among subjects with alcohol dependence. J Neural Transm (Vienna). 2007;114(4):469–72.](https://www.zotero.org/google-docs/?jPUPOW)

46b. Lin, E., Chen, P. S., Chang, H. H., Gean, P. W., Tsai, H. C., Yang, Y. K., & Lu, R. B. (2009). Interaction of serotonin-related genes affects short-term antidepressant response in major depressive disorder. Progress in neuro-psychopharmacology & biological psychiatry, 33(7), 1167–1172.

46c. Fang, L., Zhou, C., Bai, S., Huang, C., Pan, J., Wang, L., Wang, X., Mao, Q., Sun, L., & Xie, P. (2015). The C825T Polymorphism of the G-Protein β3 Gene as a Risk Factor for Depression: A Meta-Analysis. PloS one, 10(7), e0132274.

47. [Samaan Z, Anand SS, Anand S, Zhang X, Desai D, Rivera M, et al. The protective effect of the obesity-associated rs9939609 A variant in fat mass- and obesity-associated gene on depression. Mol Psychiatry. 2013 Dec;18(12):1281–6.](https://www.zotero.org/google-docs/?jPUPOW)

47b. Milaneschi, Y., Lamers, F., Mbarek, H., Hottenga, J. J., Boomsma, D. I., & Penninx, B. W. (2014). The effect of FTO rs9939609 on major depression differs across MDD subtypes. Molecular psychiatry, 19(9), 960–962.

[48. Jin C, Xu W, Yuan J, Wang G, Cheng Z. Meta-analysis of association between the -1438A/G (rs6311) polymorphism of the serotonin 2A receptor gene and major depressive disorder. Neurological Research. 2013 Jan;35(1):7–14.](https://www.zotero.org/google-docs/?jPUPOW)

48b. Viikki, M., Huuhka, K., Leinonen, E., Illi, A., Setälä-Soikkeli, E., Huuhka, M., Mononen, N., Lehtimäki, T., & Kampman, O. (2011). Interaction between two HTR2A polymorphisms and gender is associated with treatment response in MDD. Neuroscience letters, 501(1), 20–24.

[49. Li X-B, Wang J, Xu A-D, Huang J-M, Meng L-Q, Huang R-Y, et al. Apolipoprotein E polymorphisms increase the risk of post-stroke depression. Neural Regen Res. 2016 Nov;11(11):1790–6.](https://www.zotero.org/google-docs/?jPUPOW)

49b. Chagnon, Y. C., Potvin, O., Hudon, C., & Préville, M. (2015). DNA methylation and single nucleotide variants in the brain-derived neurotrophic factor (BDNF) and oxytocin receptor (OXTR) genes are associated with anxiety/depression in older women. Frontiers in genetics, 6, 230.

50[. López-León S, Janssens ACJW, González-Zuloeta Ladd AM, Del-Favero J, Claes SJ, Oostra BA, et al. Meta-analyses of genetic studies on major depressive disorder. Mol Psychiatry. 2008 Aug;13(8):772–85.](https://www.zotero.org/google-docs/?jPUPOW)

50b. Wray, N. R., James, M. R., Dumenil, T., Handoko, H. Y., Lind, P. A., Montgomery, G. W., & Martin, N. G. (2008). Association study of candidate variants of COMT with neuroticism, anxiety and depression. American journal of medical genetics. Part B, Neuropsychiatric genetics: the official publication of the International Society of Psychiatric Genetics, 147B(7), 1314–1318.

50c. Wang, M., Ma, Y., Yuan, W., Su, K., & Li, M. D. (2016). Meta-Analysis of the COMT Val158Met Polymorphism in Major Depressive Disorder: Effect of Ethnicity. Journal of neuroimmune pharmacology : the official journal of the Society on NeuroImmune Pharmacology, 11(3), 434–445.

51[. Kishi T, Yoshimura R, Fukuo Y, Okochi T, Matsunaga S, Umene-Nakano W, et al. The serotonin 1A receptor gene confer susceptibility to mood disorders: results from an extended meta-analysis of patients with major depression and bipolar disorder. Eur Arch Psychiatry Clin Neurosci. 2013 Mar;263(2):105–18.](https://www.zotero.org/google-docs/?jPUPOW)

51b. Baune, B. T., Hohoff, C., Roehrs, T., Deckert, J., Arolt, V., & Domschke, K. (2008). Serotonin receptor 1A -1019C/G variant: impact on antidepressant pharmacoresponse in melancholic depression?. Neuroscience letters, 436(2), 111–115.

52[. Binder EB, Owens MJ, Liu W, Deveau TC, Rush AJ, Trivedi MH, et al. Association of polymorphisms in genes regulating the corticotropin-releasing factor system with antidepressant treatment response. Arch Gen Psychiatry. 2010 Apr;67(4):369–79.](https://www.zotero.org/google-docs/?jPUPOW)

53. Xu, Z., Zhang, Z., Shi, Y., Pu, M., Yuan, Y., Zhang, X., Li, L., & Reynolds, G. P. (2012). Influence and interaction of genetic polymorphisms in the serotonin system and life stress on antidepressant drug response. Journal of psychopharmacology (Oxford, England), 26(3), 349–359.

[53b. Kao W-T, Yang M-C, Lung F-W. Association between HTR1B alleles and suicidal ideation in individuals with major depressive disorder. Neuroscience Letters. 2017 Jan;638:204–10.](https://www.zotero.org/google-docs/?jPUPOW)

[54. Papiol S, Arias B, Gastó C, Gutiérrez B, Catalán R, Fañanás L. Genetic variability at HPA axis in major depression and clinical response to antidepressant treatment. J Affect Disord. 2007 Dec;104(1–3):83–90.](https://www.zotero.org/google-docs/?jPUPOW)

[55. Schumacher J, Jamra RA, Becker T, Ohlraun S, Klopp N, Binder EB, et al. Evidence for a Relationship Between Genetic Variants at the Brain-Derived Neurotrophic Factor (BDNF) Locus and Major Depression. Biological Psychiatry. 2005 Aug;58(4):307–14.](https://www.zotero.org/google-docs/?jPUPOW)

55b. Verhagen, M., van der Meij, A., van Deurzen, P. A., Janzing, J. G., Arias-Vásquez, A., Buitelaar, J. K., & Franke, B. (2010). Meta-analysis of the BDNF Val66Met polymorphism in major depressive disorder: effects of gender and ethnicity. Molecular psychiatry, 15(3), 260–271.

56. Huang J, Perlis RH, Lee PH, Rush AJ, Fava M, Sachs GS, et al. Cross-Disorder Genomewide Analysis of Schizophrenia, Bipolar Disorder, and Depression. AJP. 2010 Oct;167(10):1254–63.

57. Sullivan PF, de Geus EJC, Willemsen G, James MR, Smit JH, Zandbelt T, et al. Genome-wide association for major depressive disorder: a possible role for the presynaptic protein piccolo. Mol Psychiatry. 2009 Apr;14(4):359–75.

58. Lewis CM, Ng MY, Butler AW, Cohen-Woods S, Uher R, Pirlo K, et al. Genome-Wide Association Study of Major Recurrent Depression in the U.K. Population. AJP. 2010 Aug;167(8):949–57.

59. Muglia P, Tozzi F, Galwey NW, Francks C, Upmanyu R, Kong XQ, et al. Genome-wide association study of recurrent major depressive disorder in two European case-control cohorts. Mol Psychiatry. 2010 Jun;15(6):589–601.

60. Rietschel M, Mattheisen M, Frank J, Treutlein J, Degenhardt F, Breuer R, et al. Genome-wide association-, replication-, and neuroimaging study implicates HOMER1 in the etiology of major depression. Biol Psychiatry. 2010 Sep 15;68(6):578–85.

61. Kohli MA, Lucae S, Saemann PG, Schmidt MV, Demirkan A, Hek K, et al. The neuronal transporter gene SLC6A15 confers risk to major depression. Neuron. 2011 Apr 28;70(2):252–65.

62. Shi J, Potash JB, Knowles JA, Weissman MM, Coryell W, Scheftner WA, et al. Genome-wide association study of recurrent early-onset major depressive disorder. Mol Psychiatry. 2011 Feb;16(2):193–201.

63. Shyn SI, Shi J, Kraft JB, Potash JB, Knowles JA, Weissman MM, et al. Novel loci for major depression identified by genome-wide association study of Sequenced Treatment Alternatives to Relieve Depression and meta-analysis of three studies. Mol Psychiatry. 2011 Feb;16(2):202–15.

64. Wray NR, Pergadia ML, Blackwood DHR, Penninx BWJH, Gordon SD, Nyholt DR, et al. Genome-wide association study of major depressive disorder: new results, meta-analysis, and lessons learned. Mol Psychiatry. 2012 Jan;17(1):36–48.

65. Terracciano A, Tanaka T, Sutin AR, Sanna S, Deiana B, Lai S, et al. Genome-wide association scan of trait depression. Biol Psychiatry. 2010 Nov 1;68(9):811–7.

66. Major Depressive Disorder Working Group of the Psychiatric GWAS Consortium, Ripke S, Wray NR, Lewis CM, Hamilton SP, Weissman MM, et al. A mega-analysis of genome-wide association studies for major depressive disorder. Mol Psychiatry. 2013 Apr;18(4):497–511.

67. CONVERGE consortium. Sparse whole-genome sequencing identifies two loci for major depressive disorder. Nature. 2015 Jul 30;523(7562):588–91.

68. Aragam N, Wang K-S, Pan Y. Genome-wide association analysis of gender differences in major depressive disorder in the Netherlands NESDA and NTR population-based samples. Journal of Affective Disorders. 2011 Oct;133(3):516–21.
