# Supplementary material for: Body mass index interacts with a genetic-risk score for depression increasing the risk of the disease in high-susceptibility individuals
Source: Transl Psychiatry. 2022 Jan 24;12:30. doi: 10.1038/s41398-022-01783-7 (PMC8786870; doi:10.1038/s41398-022-01783-7)
Supplement: Supplementary file 3 — Supplementary Table 1b [file 41398_2022_1783_MOESM3_ESM.docx]

**Table S1b**. Candidate genes and number of genes analyzed per selection category.

| **Category** | **n** |
| --- | --- |
| *1) High-likelihood candidate genes for depression based on literature and previous genotyping studies conducted by our research group* | 17 |
| *MTHFR (43), TPH1 (44), TPH1/SAAL1 (45), BDNF (55), GNB3 (46), ESD/HTR2A (45), HTR2A (48), FTO (47), CRHR1/LINC02210-CRHR1 (45), APOE (49), COMT (50), HTR1A (51), CRHBP/AGGF1 (52), HTR1B (53), CRHR2 (54)* |  |
| *2) Genes reported in previous GWAS* | 57 |
| *TMTC2/SLC6A15 (61), LINC01568/LOC101928035 (64), SYT4/LINC01478 (64), C3orf70/EHHADH-AS1 (64), ITGB1/NRP1 (65), LINC00443/FAM155A (65), RORA (65), CDH13 (65), SLFN12L/SLFN14 (65), LINC01884/KLHL29 (65), LINC02223/CDH18 (65), GRM8 (65), LINC00838/PARD3 (57), PCLO (57), ETAA1/LINC01812 (63), GRM7 (63), LINC00578 (63), MKLN1 (63), ATP6V1B2 (63), ATXN7L2 (62), GNAI3 (62), LOC643542 (62), TOGARAM2/C2orf71 (62), D21S2088E/LINC01689 (62), FHIT/PTPRG (62), ZCCHC14 (66), PARP15 (66), DVL3 (66), CPM/CPSF6 (60), HOMER1/PAPD4 (60), DNAJC12/SIRT1 (67), PARP11/CCND2-AS1 (59), LINC01031/NONE (58), VCAN/HAPLN1 (58), SHC4 (68), LAPTM4A/SDC1 (68), IFT88 (56)* |  |
| *3) Genes associated with depression-related pathways* | 15 |
| *Signal transduction pathways (IFT88, CRHBP, HTR1A, APOE, ATP6V1B2, BDNF, ITGB1, NRP1, GNB3) (71,72,74); extracellular organization pathways (VCAN, HAPLN1, ITGB1) (71); circadian rhythm (TPH1, RORA, SIRT1) (69,73); others -PI3K-Akt signaling, melatonin metabolism and effects, biogenic amine synthesis, CHL1 interactions- (BDNF, ITGB1, GNB3, SIRT1, APOE, TPH1, COMT, NRP1) (25,70,72,74)* |  |
