# Supplementary material for: Body mass index interacts with a genetic-risk score for depression increasing the risk of the disease in high-susceptibility individuals
Source: Transl Psychiatry. 2022 Jan 24;12:30. doi: 10.1038/s41398-022-01783-7 (PMC8786870; doi:10.1038/s41398-022-01783-7)
Supplement: Supplementary file 4 — Supplementary Table 2 [file 41398_2022_1783_MOESM4_ESM.docx]

**Table S2.** Over-representated pathways from Reactome and WikiPathways databases

| **source** | **term_name** | **term_id** | **adjusted_p_value** | **term_size** | **intersection_size** | **intersections** |
| --- | --- | --- | --- | --- | --- | --- |
| REAC | Signaling by Receptor Tyrosine Kinases | REAC:R-HSA-9006934 | 0.000775715232103108000 | 455 | 5 | APOE,ATP6V1B2,BDNF,ITGB1,NRP1 |
| REAC | ECM proteoglycans | REAC:R-HSA-3000178 | 0.00155769475416025 | 75 | 3 | VCAN,HAPLN1,ITGB1 |
| REAC | CHL1 interactions | REAC:R-HSA-447041 | 0.00189391004724318 | 9 | 2 | ITGB1,NRP1 |
| REAC | Signal Transduction | REAC:R-HSA-162582 | 0.00203823721651071 | 2717 | 9 | IFT88,CRHBP,HTR1A,APOE,ATP6V1B2,BDNF,ITGB1,NRP1,GNB3 |
| REAC | Signal transduction by L1 | REAC:R-HSA-445144 | 0.00995942989008595 | 20 | 2 | ITGB1,NRP1 |
| REAC | Circadian Clock | REAC:R-HSA-400253 | 0.0212071841885873 | 67 | 2 | RORA,SIRT1 |
| REAC | Extracellular matrix organization | REAC:R-HSA-1474244 | 0.028044036899063 | 298 | 2 | VCAN,HAPLN1 |
| WP | Sudden Infant Death Syndrome (SIDS) Susceptibility Pathways | WP:WP706 | 1,39E-08 | 158 | 5 | TPH1,RORA,HTR1A,BDNF,GNB3 |
| WP | Biogenic Amine Synthesis | WP:WP550 | 0.00020764585008238 | 15 | 2 | TPH1,COMT |
| WP | Circadian rhythm related genes | WP:WP3594 | 0.000701571597879841 | 201 | 3 | TPH1,RORA,SIRT1 |
| WP | Melatonin metabolism and effects | WP:WP3298 | 0.00377076918850939 | 37 | 2 | SIRT1,APOE |
| WP | PI3K-Akt Signaling Pathway | WP:WP4172 | 0.0464345695331761 | 340 | 3 | BDNF,ITGB1,GNB3 |
