# Supplementary material for: Body mass index interacts with a genetic-risk score for depression increasing the risk of the disease in high-susceptibility individuals
Source: Transl Psychiatry. 2022 Jan 24;12:30. doi: 10.1038/s41398-022-01783-7 (PMC8786870; doi:10.1038/s41398-022-01783-7)
Supplement: Supplementary file 5 — Supplementary Table 3 [file 41398_2022_1783_MOESM5_ESM.docx]

**Table S3**. Demographic characteristics of the study population by experimental condition.

|  | | **MDD-cases** | **Controls** | **Effect size** | **p-value** |
| --- | --- | --- | --- | --- | --- |
|  |  | n=104 | n=1546 |  |  |
| Age (years) | | 53.51 (14.99) | 51.57 (14.50) | 0.132 | 0.187 |
| Sex (male/female) | | 37/67 | 586/960 | 0.12 | 0.636 |
| BMI (kg/m^2^) | | 27.99 (5.24) | 26.80  (4.52) | 0.244 | **0.026** |
| GRS | | 22.38 (2.91) | 20.71  (2.94) | 0.571 | **1.02** **x10^-7^** |
| Province | Barcelona  Bilbao  Granada  Jaen  Malaga  Valladolid  Zaragoza | 9  7  16  23  25  14  10 | 132  181  238  230  211  293  261 | 0.103 | **0.008** |

Abbreviations: BMI, body mass index; GRS, genetic risk score. Data are expressed as mean (standard deviation). p-values of the categorical variables Sex and Province were obtained after performing a χ² test. Effect sizes were reported as Cohen’s d and Cramer’s V for quantitative and qualitative variables, respectively.
