# Supplementary material for: Body mass index interacts with a genetic-risk score for depression increasing the risk of the disease in high-susceptibility individuals
Source: Transl Psychiatry. 2022 Jan 24;12:30. doi: 10.1038/s41398-022-01783-7 (PMC8786870; doi:10.1038/s41398-022-01783-7)
Supplement: Supplementary file 6 — Supplementary Table 4 [file 41398_2022_1783_MOESM6_ESM.docx]

**Table S4.** SNPs-based association test on MDD status

| **SNP** | **Effect Allele** | **OR** | **beta** | **SE** | **ci.lo** | **ci.up** | **Wald_Test** | ***P-*value** | **FDR** |
| --- | --- | --- | --- | --- | --- | --- | --- | --- | --- |
| (Intercept) | - | 0.01 | -5.07 | 0.82 | 0.00 | 0.03 | -6.17 | < 0.0001 | < 0.0001 |
| rs1801133 | A | 1.02 | 0.02 | 0.16 | 0.75 | 1.38 | 0.10 | 0.92 | 1.00 |
| rs12049330 | G | 1.28 | 0.25 | 0.23 | 0.81 | 2.03 | 1.07 | 0.28 | 0.67 |
| **rs6537837** | **T** | **0.59** | **-0.53** | **0.24** | **0.37** | **0.95** | **-2.16** | **0.03** | **0.35** |
| rs606149 | T | 0.80 | -0.23 | 0.16 | 0.58 | 1.09 | -1.42 | 0.16 | 0.50 |
| rs7565124 | A | 1.04 | 0.04 | 0.17 | 0.74 | 1.45 | 0.21 | 0.83 | 0.99 |
| rs1449984 | G | 0.99 | -0.01 | 0.17 | 0.71 | 1.37 | -0.08 | 0.94 | 1.00 |
| rs882632 | T | 1.29 | 0.26 | 0.16 | 0.94 | 1.78 | 1.59 | 0.11 | 0.45 |
| rs724568 | C | 0.85 | -0.16 | 0.16 | 0.62 | 1.17 | -0.98 | 0.32 | 0.67 |
| rs9870680 | T | 0.81 | -0.21 | 0.16 | 0.59 | 1.11 | -1.32 | 0.19 | 0.56 |
| rs10514718 | G | 1.55 | 0.44 | 0.29 | 0.88 | 2.73 | 1.52 | 0.13 | 0.49 |
| rs2173763 | G | 1.38 | 0.32 | 0.27 | 0.81 | 2.35 | 1.18 | 0.24 | 0.65 |
| rs644695 | G | 0.96 | -0.04 | 0.19 | 0.66 | 1.41 | -0.21 | 0.83 | 0.99 |
| rs1969253 | C | 1.12 | 0.12 | 0.16 | 0.82 | 1.53 | 0.74 | 0.46 | 0.84 |
| rs7647854 | G | 1.07 | 0.07 | 0.21 | 0.71 | 1.61 | 0.31 | 0.75 | 0.99 |
| **rs349475** | **T** | **1.58** | **0.46** | **0.16** | **1.16** | **2.16** | **2.90** | **0.003** | **0.11** |
| rs6295 | C | 1.17 | 0.16 | 0.15 | 0.87 | 1.59 | 1.04 | 0.30 | 0.67 |
| rs10473984 | T | 1.42 | 0.35 | 0.33 | 0.75 | 2.68 | 1.07 | 0.28 | 0.67 |
| rs7713917 | G | 1.05 | 0.05 | 0.16 | 0.77 | 1.43 | 0.31 | 0.76 | 0.99 |
| **rs310501** | **G** | **1.55** | **0.44** | **0.17** | **1.12** | **2.15** | **2.63** | **0.01** | **0.16** |
| rs6296 | G | 0.69 | -0.36 | 0.19 | 0.48 | 1.01 | -1.89 | 0.06 | 0.35 |
| rs2270007 | G | 1.42 | 0.35 | 0.20 | 0.95 | 2.11 | 1.73 | 0.08 | 0.39 |
| rs2715148 | C | 1.33 | 0.28 | 0.15 | 0.99 | 1.78 | 1.88 | 0.06 | 0.35 |
| rs17864092 | C | 0.89 | -0.11 | 0.23 | 0.57 | 1.40 | -0.50 | 0.62 | 0.98 |
| rs10265216 | A | 1.00 | 0.00 | 0.16 | 0.74 | 1.36 | 0.00 | 1.00 | 1.00 |
| rs1106634 | A | 1.19 | 0.17 | 0.22 | 0.77 | 1.83 | 0.77 | 0.44 | 0.83 |
| rs11009175 | A | 1.07 | 0.06 | 0.21 | 0.70 | 1.62 | 0.30 | 0.77 | 0.99 |
| rs1780436 | A | 1.19 | 0.17 | 0.16 | 0.88 | 1.62 | 1.11 | 0.27 | 0.67 |
| rs12415800 | A | 2.02 | 0.70 | 0.55 | 0.69 | 5.89 | 1.29 | 0.20 | 0.56 |
| **rs1800532** | **T** | **1.48** | **0.39** | **0.19** | **1.02** | **2.15** | **2.04** | **0.04** | **0.35** |
| rs623580 | A | 1.15 | 0.14 | 0.20 | 0.77 | 1.72 | 0.70 | 0.49 | 0.84 |
| rs6265 | T | 0.93 | -0.08 | 0.19 | 0.64 | 1.35 | -0.39 | 0.70 | 0.99 |
| rs4238010 | G | 1.03 | 0.03 | 0.30 | 0.57 | 1.87 | 0.11 | 0.92 | 1.00 |
| rs5443 | T | 1.04 | 0.04 | 0.16 | 0.76 | 1.42 | 0.25 | 0.81 | 0.99 |
| rs9943849 | T | 1.41 | 0.34 | 0.18 | 0.98 | 2.02 | 1.88 | 0.06 | 0.35 |
| rs1545843 | G | 1.11 | 0.11 | 0.15 | 0.83 | 1.50 | 0.71 | 0.48 | 0.84 |
| rs7326068 | A | 1.43 | 0.36 | 0.19 | 0.99 | 2.07 | 1.91 | 0.06 | 0.35 |
| rs9526236 | C | 1.25 | 0.23 | 0.16 | 0.92 | 1.71 | 1.43 | 0.15 | 0.50 |
| rs6311 | T | 1.16 | 0.15 | 0.15 | 0.86 | 1.56 | 0.98 | 0.33 | 0.67 |
| rs1927745 | A | 0.94 | -0.06 | 0.20 | 0.64 | 1.38 | -0.30 | 0.76 | 0.99 |
| rs8023445 | C | 0.89 | -0.11 | 0.27 | 0.52 | 1.52 | -0.42 | 0.68 | 0.99 |
| rs12912233 | T | 1.30 | 0.27 | 0.16 | 0.96 | 1.77 | 1.71 | 0.09 | 0.39 |
| rs9939609 | A | 0.98 | -0.02 | 0.16 | 0.71 | 1.34 | -0.15 | 0.88 | 1.00 |
| rs12446956 | C | 1.10 | 0.09 | 0.24 | 0.69 | 1.76 | 0.40 | 0.69 | 0.99 |
| rs10514585 | A | 0.99 | -0.01 | 0.18 | 0.70 | 1.41 | -0.03 | 0.97 | 1.00 |
| rs9646303 | G | 0.67 | -0.40 | 0.23 | 0.42 | 1.05 | -1.74 | 0.08 | 0.39 |
| rs8070473 | T | 0.89 | -0.11 | 0.18 | 0.63 | 1.28 | -0.61 | 0.54 | 0.90 |
| rs110402 | A | 0.99 | -0.01 | 1.32 | 0.08 | 13.06 | -0.01 | 0.99 | 1.00 |
| rs242924 | T | 1.10 | 0.10 | 1.32 | 0.08 | 14.52 | 0.07 | 0.94 | 1.00 |
| **rs242939** | **C** | **0.35** | **-1.05** | **0.49** | **0.14** | **0.91** | **-2.16** | **0.03** | **0.35** |
| rs1876828 | T | 0.90 | -0.10 | 0.20 | 0.61 | 1.34 | -0.51 | 0.61 | 0.98 |
| rs12457996 | C | 0.84 | -0.18 | 0.19 | 0.57 | 1.22 | -0.92 | 0.36 | 0.70 |
| rs17077540 | G | 1.01 | 0.01 | 0.22 | 0.65 | 1.57 | 0.04 | 0.97 | 1.00 |
| rs429358 | C | 1.30 | 0.26 | 0.26 | 0.78 | 2.17 | 1.02 | 0.31 | 0.67 |
| rs7412 | T | 0.89 | -0.12 | 0.35 | 0.45 | 1.76 | -0.33 | 0.74 | 0.99 |
| rs2828520 | G | 0.96 | -0.04 | 0.16 | 0.70 | 1.32 | -0.24 | 0.81 | 0.99 |
| rs4680 | A | 1.25 | 0.22 | 0.16 | 0.92 | 1.70 | 1.43 | 0.15 | 0.50 |

These results come from a single logistic regression model with all SNPs as covariates. Abbreviations: SNP, Single Nucleotide Polymorphism; OR, odds ratio; SE, standard error; ci.lo, 95 % lower confidence interval; ci.up, 95 % upper confidence interval and FDR, False Discovery Rate.
