# Supplementary material for: Body mass index interacts with a genetic-risk score for depression increasing the risk of the disease in high-susceptibility individuals
Source: Transl Psychiatry. 2022 Jan 24;12:30. doi: 10.1038/s41398-022-01783-7 (PMC8786870; doi:10.1038/s41398-022-01783-7)
Supplement: Supplementary file 7 — Supplementary Table 5 [file 41398_2022_1783_MOESM7_ESM.docx]

**Table S5.** Average statistics for model improvement with addition of genetic and non-genetic risk factors for MDD after implementation of 5-fold cross-validation procedure.

|  | **Initial model: Model 1**  **Final model: Model 2** | **Initial model: Model 1**  **Final model: Model 3** | **Initial model: Model 3**  **Final model: Model 4** | **Initial model: Model 3**  **Final model: Model 5** | **Initial model: Model 4**  **Final model: Model 5** |
| --- | --- | --- | --- | --- | --- |
| NRI | 0.01 (-0.076,0.128) | 0.10 (-0.188, 0.288) | -0.002 (-0.096, 0.096) | 0.09 (-0.074, 0.274) | 0.10 (-0.058, 0.266) |
| NRI *P-*value | 0.52 | 0.05 | 0.46 | **3e-03** | **0.02** |
| cfNRI | 0.17 (-0.256, 0.614) | 0.42 (-0.005, 0.85) | 0.14 (-5.71, 0.588) | 0.17 (-0.26, 0.60) | 0.19 (-0.234, 0.63) |
| cfNRI *P-*value | 0.19 | **2e-04** | 0.28 | 0.43 | 0.14 |
| IDI | 8e-04 (-0.01, 0.006) | 0.016 (2e-04, 0.036 | 8e-04 (-0.008, 0.02) | 0.01 (-0.007, 0.038) | 0.01 (-0.005, 0.03) |
| IDI *P-*value | 0.18 | **1e-04** | 0.2 | 0.06 | **3e-03** |

Model 1 (Sex+Age+Province); Model 2 (Sex+Age+Province+BMI); Model 3 (Sex+Age+Province+GRS), Model 4 (Sex+Age+Province+GRS+BMI) and Model 5 (Sex+Age+Province+GRS*BMI). Abbreviations: NRI, net reclassification improvement; cfNRI, category-free NRI; IDI, integrated discrimination improvement. The 95% confidence intervals are shown in parentheses.
