# Supplementary material for: Body mass index interacts with a genetic-risk score for depression increasing the risk of the disease in high-susceptibility individuals
Source: Transl Psychiatry. 2022 Jan 24;12:30. doi: 10.1038/s41398-022-01783-7 (PMC8786870; doi:10.1038/s41398-022-01783-7)
Supplement: Supplementary file 8 — Supplementary Table 6 [file 41398_2022_1783_MOESM8_ESM.docx]

**Table S6.** Average AUC obtained for each model in the test sample after the implementation of 5-fold cross-validation procedure.

|  | **AUC average** |
| --- | --- |
| Model 1 | 0.56 (0.43, 0.70) |
| Model 2 | 0.57 (0.44, 0.70) |
| Model 3 | 0.63 (0.51, 0.76) |
| Model 4 | 0.63 (0.51, 76) |
| Model 5 | **0.65 (0.54, 0.78)** |

Model 1 (Sex+Age+Province); Model 2 (Sex+Age+Province+BMI); Model 3 (Sex+Age+Province+GRS), Model 4 (Sex+Age+Province+GRS+BMI) and Model 5 (Sex+Age+Province+GRS*BMI). Abbreviations: AUC, area under the curve of the receiver operator characteristic curve
