# Supplementary material for: Body mass index interacts with a genetic-risk score for depression increasing the risk of the disease in high-susceptibility individuals
Source: Transl Psychiatry. 2022 Jan 24;12:30. doi: 10.1038/s41398-022-01783-7 (PMC8786870; doi:10.1038/s41398-022-01783-7)
Supplement: Supplementary file 9 — Supplementary Table 7 [file 41398_2022_1783_MOESM9_ESM.docx]

**Table S7.** SNP-based association tests on BMI.

| **SNP** | **Effect Allele** | **Beta** | **SE** | **ci.lo** | **ci.up** | **t-value** | ***P-*value** | **FDR** |
| --- | --- | --- | --- | --- | --- | --- | --- | --- |
| rs10473984 | T | -0.13 | 0.35 | -0.83 | 0.56 | -0.38 | 0.71 | 0.85 |
| rs11009175 | A | 0.04 | 0.21 | -0.37 | 0.46 | 0.2 | 0.84 | 0.85 |
| rs110402 | A | 0.16 | 0.16 | -0.14 | 0.47 | 1.06 | 0.29 | 0.85 |
| rs1106634 | A | 0.18 | 0.23 | -0.27 | 0.64 | 0.79 | 0.43 | 0.85 |
| rs12049330 | G | 0.25 | 0.21 | -0.17 | 0.66 | 1.17 | 0.24 | 0.85 |
| rs12415800 | A | 0.44 | 0.76 | -1.06 | 1.93 | 0.57 | 0.57 | 0.85 |
| rs12446956 | C | 0.06 | 0.24 | -0.41 | 0.53 | 0.24 | 0.81 | 0.85 |
| **rs12457996** | **C** | **-0.42** | **0.19** | **-0.8** | **-0.05** | **-2.2** | **0.03** | **0.82** |
| rs12912233 | T | 0.03 | 0.15 | -0.27 | 0.33 | 0.19 | 0.85 | 0.85 |
| rs17077540 | G | 0.15 | 0.21 | -0.28 | 0.57 | 0.68 | 0.50 | 0.85 |
| rs1800532 | T | 0.26 | 0.16 | -0.05 | 0.57 | 1.64 | 0.10 | 0.85 |
| rs1876828 | T | -0.10 | 0.16 | -0.42 | 0.22 | -0.6 | 0.55 | 0.85 |
| rs2173763 | G | 0.24 | 0.29 | -0.34 | 0.81 | 0.81 | 0.42 | 0.85 |
| rs242924 | T | 0.18 | 0.15 | -0.13 | 0.48 | 1.15 | 0.25 | 0.85 |
| rs242939 | C | 0.06 | 0.30 | -0.53 | 0.66 | 0.21 | 0.83 | 0.85 |
| rs2715148 | C | -0.08 | 0.15 | -0.37 | 0.22 | -0.51 | 0.61 | 0.85 |
| rs310501 | G | -0.06 | 0.17 | -0.39 | 0.27 | -0.38 | 0.70 | 0.85 |
| rs349475 | T | -0.04 | 0.16 | -0.36 | 0.28 | -0.26 | 0.79 | 0.85 |
| rs429358 | C | -0.16 | 0.27 | -0.69 | 0.38 | -0.57 | 0.57 | 0.85 |
| rs4680 | A | 0.10 | 0.15 | -0.2 | 0.4 | 0.67 | 0.50 | 0.85 |
| rs5443 | T | 0.08 | 0.16 | -0.24 | 0.39 | 0.48 | 0.63 | 0.85 |
| rs6265 | T | -0.05 | 0.18 | -0.41 | 0.31 | -0.26 | 0.80 | 0.85 |
| rs6295 | C | 0.11 | 0.15 | -0.19 | 0.41 | 0.72 | 0.47 | 0.85 |
| rs7326068 | A | 0.32 | 0.20 | -0.07 | 0.71 | 1.63 | 0.10 | 0.85 |
| rs7412 | T | 0.17 | 0.33 | -0.48 | 0.82 | 0.51 | 0.61 | 0.85 |
| rs7647854 | G | 0.11 | 0.21 | -0.29 | 0.52 | 0.55 | 0.58 | 0.85 |
| rs8023445 | C | -0.07 | 0.26 | -0.58 | 0.45 | -0.25 | 0.80 | 0.85 |
| rs8070473 | T | -0.03 | 0.17 | -0.37 | 0.3 | -0.2 | 0.84 | 0.85 |
| rs882632 | T | -0.08 | 0.17 | -0.41 | 0.25 | -0.46 | 0.64 | 0.85 |
| rs9943849 | T | 0.27 | 0.19 | -0.1 | 0.64 | 1.42 | 0.16 | 0.85 |
| rs10473984 | T | -0.13 | 0.35 | -0.83 | 0.56 | -0.38 | 0.71 | 0.85 |
| rs11009175 | A | 0.04 | 0.21 | -0.37 | 0.46 | 0.2 | 0.84 | 0.85 |
| rs110402 | A | 0.16 | 0.16 | -0.14 | 0.47 | 1.06 | 0.29 | 0.85 |
| rs1106634 | A | 0.18 | 0.23 | -0.27 | 0.64 | 0.79 | 0.43 | 0.85 |
| rs12049330 | G | 0.25 | 0.21 | -0.17 | 0.66 | 1.17 | 0.24 | 0.85 |
| rs12415800 | A | 0.44 | 0.76 | -1.06 | 1.93 | 0.57 | 0.57 | 0.85 |
| rs12446956 | C | 0.06 | 0.24 | -0.41 | 0.53 | 0.24 | 0.81 | 0.85 |
| rs12457996 | C | -0.42 | 0.19 | -0.8 | -0.05 | -2.2 | 0.03 | 0.82 |
| rs12912233 | T | 0.03 | 0.15 | -0.27 | 0.33 | 0.19 | 0.85 | 0.85 |
| rs17077540 | G | 0.15 | 0.21 | -0.28 | 0.57 | 0.68 | 0.50 | 0.85 |
| rs1800532 | T | 0.26 | 0.16 | -0.05 | 0.57 | 1.64 | 0.10 | 0.85 |
| rs1876828 | T | -0.10 | 0.16 | -0.42 | 0.22 | -0.6 | 0.55 | 0.85 |
| rs2173763 | G | 0.24 | 0.29 | -0.34 | 0.81 | 0.81 | 0.42 | 0.85 |
| rs242924 | T | 0.18 | 0.15 | -0.13 | 0.48 | 1.15 | 0.25 | 0.85 |
| rs242939 | C | 0.06 | 0.30 | -0.53 | 0.66 | 0.21 | 0.83 | 0.85 |
| rs2715148 | C | -0.08 | 0.15 | -0.37 | 0.22 | -0.51 | 0.61 | 0.85 |
| rs310501 | G | -0.06 | 0.17 | -0.39 | 0.27 | -0.38 | 0.70 | 0.85 |
| rs349475 | T | -0.04 | 0.16 | -0.36 | 0.28 | -0.26 | 0.79 | 0.85 |
| rs429358 | C | -0.16 | 0.27 | -0.69 | 0.38 | -0.57 | 0.57 | 0.85 |
| rs4680 | A | 0.10 | 0.15 | -0.2 | 0.4 | 0.67 | 0.50 | 0.85 |
| rs5443 | T | 0.08 | 0.16 | -0.24 | 0.39 | 0.48 | 0.63 | 0.85 |
| rs6265 | T | -0.05 | 0.18 | -0.41 | 0.31 | -0.26 | 0.80 | 0.85 |
| rs6295 | C | 0.11 | 0.15 | -0.19 | 0.41 | 0.72 | 0.47 | 0.85 |
| rs7326068 | A | 0.32 | 0.20 | -0.07 | 0.71 | 1.63 | 0.10 | 0.85 |
| rs7412 | T | 0.17 | 0.33 | -0.48 | 0.82 | 0.51 | 0.61 | 0.85 |

Abbreviations: SNP, Single Nucleotide Polymorphism; βeta, Beta effect obtained under an additive genetic model of inheritance; SE, standard error, ci.lo, 95 % lower confidence interval; ci.up, 95 % upper confidence interval and FDR, false discovery rate.
