# Supplementary figures and images for: Body mass index interacts with a genetic-risk score for depression increasing the risk of the disease in high-susceptibility individuals
Source: Transl Psychiatry. 2022 Jan 24;12:30. doi: 10.1038/s41398-022-01783-7 (PMC8786870; doi:10.1038/s41398-022-01783-7)

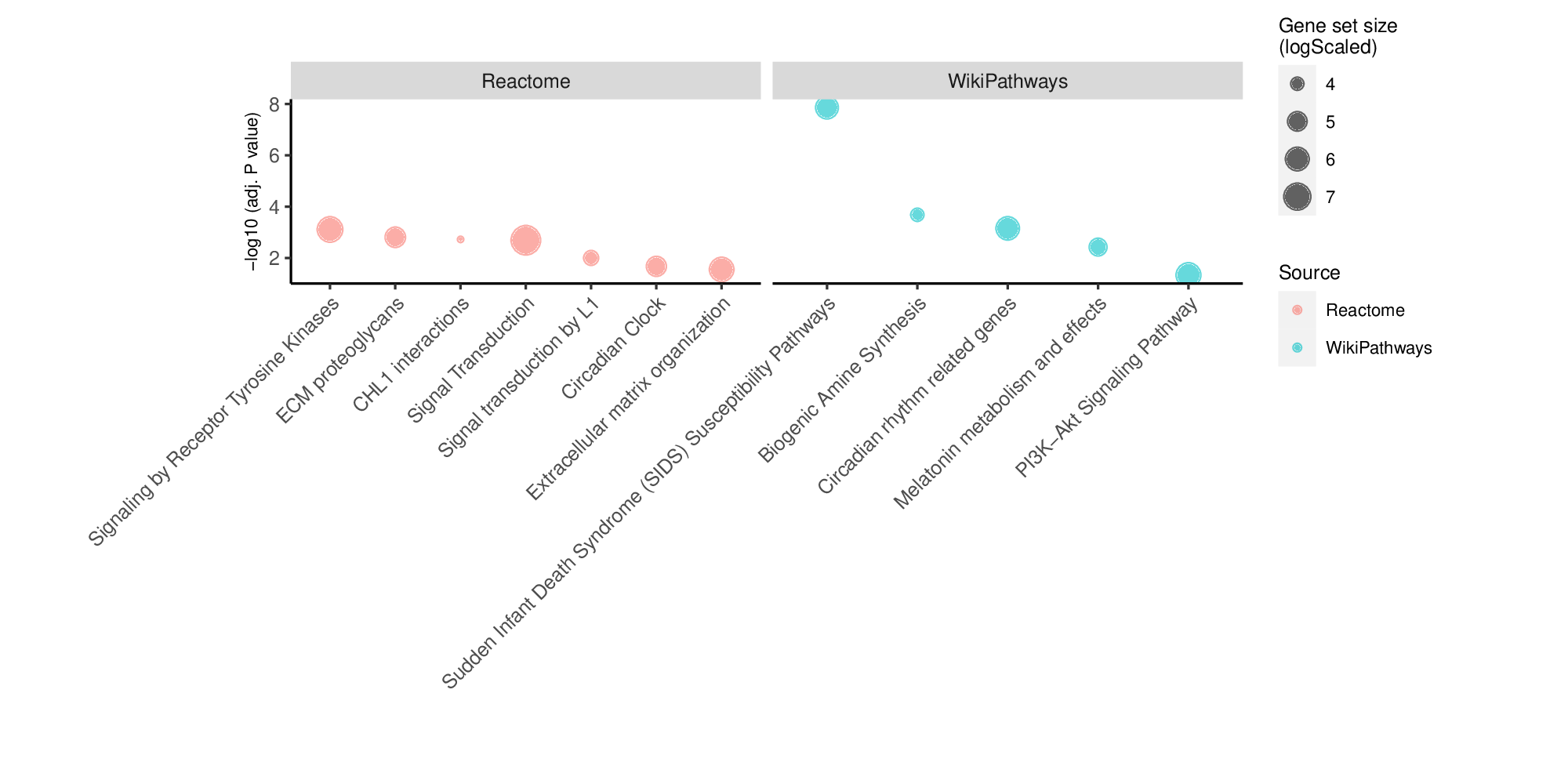

Supplement: Supplementary file 10 — Supplementary Figure 1 [file 41398_2022_1783_MOESM10_ESM.tif]

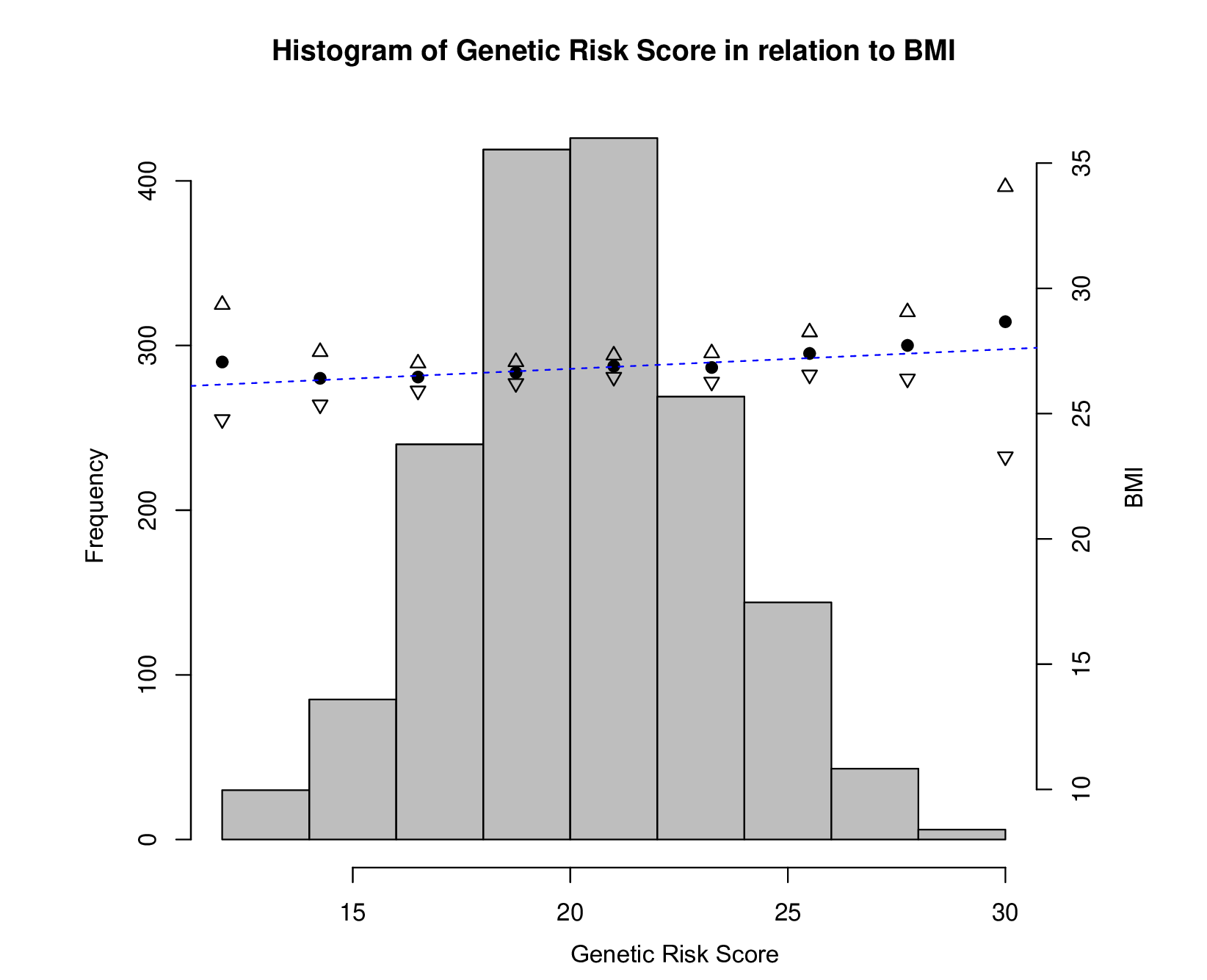

Supplement: Supplementary file 12 — Supplementary Figure 3 [file 41398_2022_1783_MOESM12_ESM.tif]
